# Editorial: A holistic and embodied approach to movement programming for health and well-being

**DOI:** 10.3389/fpubh.2025.1688972

**Published:** 2025-09-15

**Authors:** Maria Kosma

**Affiliations:** School of Kinesiology, Louisiana State University, Baton Rouge, LA, United States

**Keywords:** embodied movement, holistic health, Merleau-Ponty, preventive medicine, exercise program for health promotion, exercise science, clinical populations, health-related quality of life

## Research Topic contextualization

Based on the World Obesity Federation (1), over one billion people in the world live with obesity. Childhood and adolescent obesity levels have risen 5fold between 1975 (4%) and 2022 (20%). In the USA, about 44% of young adults are classified as obese. High obesity rates, sedentary lifestyles, poor diet, certain diseases and health conditions, socio-demographic factors, and societal pressures are associated with decreased health and wellbeing (2–5). Although physical activity can curb high obesity levels and its underlying conditions [e.g., heart disease, stroke, type II diabetes and certain types of cancer; (6, 7)], regular exercise participation is a significant challenge (8). About 27.5% of adults worldwide do not meet the recommended exercise levels. Women and people in low-income countries are significantly less active than men and those living in wealthy areas. Worryingly, about 81% of children and adolescents do not participate in moderate-to-vigorous physical activity for 60 min per day, and girls are significantly less active (85%) than boys [77.6%; (9)].

To address these public health concerns, a holistic approach to movement programming was embraced using Merleau-Ponty's *Phenomenology of Perception*. Specifically, drawing on Merleau-Ponty's philosophical underpinnings, body and mind work in unison via bodily movement to understand the world and impact health. The body is not inferior to the mind. It is not an object or a statistic like a table or a chair. Merleau-Ponty elevated the body to a subject, the *Lived Body*, that acts by throwing itself into meaningful movement significations like dancing and aerial dancing, jogging, swimming, and weight training (10–14). While kinesiologists and public health experts measure certain physiological indicators (e.g., cortisol levels) to understand different mechanisms related to the effects of exercise on health, it is impossible to simultaneously assess and monitor the cascade of changes (e.g., physiological, psychological, and cognitive) that occur prior to, during, and following an exercise program. It is recognized in this editorial that the effects of movement on health are multi-layered involving the interaction of different systems in the human organism (e.g., musculoskeletal, neuroendocrine, nervous, respiratory, circulatory, and digestive), genetics, socio-demographic and environmental aspects, psycho-social factors, and certain diseases and clinical conditions. The body and its functions are viewed holistically, whereby action requires consciousness (body as being for-itself) and its interaction with unconscious mechanisms like the autonomic nervous system (body as being in-itself) (10, 15–19).

## Research Topic purpose and aims

Based on this holistic view of bodily movement and its functions, mental health is not separate from physical health. Embodied and holistic exercise programs can affect all areas of health, including body schema, fitness, energy levels, mental and physical health, and lifestyle (13, 17, 20, 21). Therefore, the purpose of this Research Topic was to examine the effects of holistic and embodied movement programs and therapies on physical and physiological health, mental health, health-related quality of life, and lifestyle choices among different populations, including children and adolescents, young adults, and older adults. Such programs should activate all major muscle groups, including mindfulness in expression and body awareness. They can be physically demanding and derive from a variety of settings, such as exercise, physical activity, and rehabilitation. Five peer-reviewed articles are published on this Research Topic, and a brief description of each study is provided below.

## Summary of published articles on the Research Topic

A 12-week circuit training program among 25 obese adult men was effective in improving body mass index, total antioxidant status, oxidative stress index, nitric oxide, and brachial-ankle pulse wave velocity (Son et al.). The treatment group engaged in such exercises as push-ups, squats, lunges, shoulder presses, pull-ups, hip-bridges, and leg-raises three times per week at 60%−80% of heart rate reserve, whereas the control group did not participate in a structured exercise program. Circuit training can decrease oxidative stress, which tends to be high among obese individuals, and thus improve cardiovascular health.

Given the low physical activity levels among children and adolescents in Ecuador and the importance of exercise among cancer patients, a randomized controlled trial was proposed to examine the effects of a 10-week structured physical activity program (120 min per week) on health-related quality of life (e.g., physical, mental, and psychosocial health) among children and adolescents in Quito, Ecuador (Galárraga et al.). The participants will be undergoing oncological therapy or post-treatment monitoring. A follow-up assessment in week 24 will also take place.

Rural older adults in China tend to be less active than people in urban areas. Therefore, in a cross-sectional study it was shown that the promotion of physical activity participation among rural older adults is based on demographics (e.g., socio-economic status), health, exercise guidance, as well as availability and access to physical fitness facilities and programs (Zhang et al.).

Given the interest in the therapeutic effects of massage during the perioperative period, USA has exhibited the highest volume of related publications up until 2019 followed by China and Turkey (Li et al.). After 2019, China surpassed all other countries in publication growth with emphasis on acupoint massage. Strong international collaborations on the topic occurred between USA and Brazil. Massage therapy has shown to decrease anxiety and alleviate pain related to such conditions as carpal tunnel syndrome, breast cancer, cardiac surgery, and total knee arthroplasty. Examining the mechanisms related to these positive effects is an important future direction.

Given the increasing levels of depression among young adults, in a systematic review and meta-analysis the anti-depressant effects of mainly 24-style Tai Chi among young individuals aged 15–24 years were showcased (Huang et al.). The optimal reduction in depression was shown for Tai Chi programs with a 12-week duration and a training time of over 3 h/week.

## Conclusion

Based on the published studies on this Research Topic, holistic and embodied movement interventions (e.g., Tai Chi, circuit training, and massage therapy) seem to have positive effects on physical and physiological health, mental health, and health-related quality of life among various populations. Viewing the body as a subject (for-itself, in-itself, and their interplay) and acknowledging the complex relations among exercise programs, choices, and health are key to preventive medicine, treatment, healing, and wellbeing.
